# Understanding the impact of Covid-19 on the delivery and receipt of prison healthcare: an international scoping review

**DOI:** 10.1186/s40352-023-00242-9

**Published:** 2023-10-17

**Authors:** Pip Hearty, Krysia Canvin, Sue Bellass, Sarah Hampton, Nat Wright, Laura Sheard

**Affiliations:** 1 Spectrum Community Health CIC, Wakefield, UK; 2 Leeds Institute of Health Sciences, University of Leeds, Leeds, UK; 3 Department of Sport and Exercise Sciences, Manchester Metropolitan University, Manchester, UK; 4 York Trials Unit, Department of Health Sciences, University of York, York, UK; 5 Department of Health Sciences, University of York, York, UK

**Keywords:** Prison healthcare, Covid-19, Correctional healthcare, Prisons, Jail, Healthcare delivery

## Abstract

**Background:**

People being held in prison are particularly vulnerable to Covid-19 infection, as places of detention are high-risk environments for spread of infection. Due to this risk, many prisons across the globe introduced measures to reduce the risk of Covid-19 transmission. The pandemic changed almost all aspects of prison life, including prison healthcare provision. We undertook a scoping review to understand what is known about the impact of the Covid-19 pandemic on the receipt and delivery of prison healthcare. This scoping review is part of a wider mixed-methods study focusing more specifically on the impact that Covid-19 had on prison healthcare delivery in England.

**Methods:**

We conducted an international scoping review of peer-reviewed articles published between December 2019 and January 2022, across six electronic databases. We also conducted a hand search of key journals and the reference lists of included articles.

**Results:**

Twelve articles met our inclusion criteria. The articles focused primarily on prisons in high-income countries and mostly explored the impact that the pandemic had on the provision of drug treatment services. Some aspects of drug treatment services were more impacted than others, with those delivered by external providers and preparations for release particularly hindered. Whilst prison mental health services were purportedly available, there were changes regarding how these were delivered, with group therapies suspended and most consultations taking place using telehealth. The articles reported both digital and non-digital adaptations or innovations to prison healthcare services to ensure continued delivery. Collaboration between different agencies, such as the prison itself, healthcare providers, and non-governmental organisations, was key to facilitating ongoing provision of healthcare to people in prison.

**Conclusions:**

Covid-19 impacted on prison healthcare internationally, but different treatment services were affected in disparate ways, both within and between countries. The published literature concentrates on the impact on drug treatment services. Prison healthcare providers rapidly adapted their processes to attempt to maintain service provision.

**Supplementary Information:**

The online version contains supplementary material available at 10.1186/s40352-023-00242-9.

## Background

Specific groups of people are overrepresented in the prison population, including people from ethnic minorities, people from deprived backgrounds, and those with complex and multiple health needs (Fazel & Baillargeon, 2011). These groups are also likely to be disproportionately impacted by Covid-19 (Public Health England, 2020). The prison population has a higher disease burden and a higher proportion of complex health needs than communities outside the prison gates (WHO, 2019). All of the above equates to a population inherently more vulnerable to Covid-19, coupled with the risk of residing in a closed environment during a pandemic (Akiyama et al., 2020). Due to this risk, prison authorities in several countries, including England and Wales, implemented a swift lockdown in early 2020 in response to Covid-19, including suspending external visits to people in prison, reducing movement across the prison estate, and limiting the time people in prison could spend outside of their cell (Fair & Jacobson, 2021; Heard, 2021). In England and Wales, this resulted in the majority of people in prison spending almost the entire day inside their cells (Edge et al., 2021). In these early stages of the pandemic, published statements concentrated on how prepared the prison estate was for the impending pandemic (WHO, 2020). There were propositions for practical responses to avoid fatalities, such as the use of temporary cells and early release for some groups of people being held in custody (Shilson-Thomas, 2020). Other commentators pointed to the high risk for imprisoned people who had ongoing mental health concerns and the consequent potential for the disruption of mental healthcare provision (Liebrenz et al., 2020).

Published in 2021, stark evidence garnered from people in prison across 10 countries pointed towards imprisoned people being locked in cells for long periods with little or no social contact, leading to prolonged distress and isolation (Heard, 2021). The mental health and emotional wellbeing of people in prison deteriorated as confinement and segregation continued (Wainwright & Gipson, 2020). People in prison reported feeling isolated, frustrated, stressed, and worried during the pandemic (User Voice, 2020), and were locked in their cells for an average of 22.5 h per day in England (Edge et al., 2021). Physical health was also a concern for people in prison, many of whom felt that it had declined since the start of the pandemic, and this was often attributed to a lack of available physical activity, and with shortcomings in basic support services provided to people in prison (Gipson & Wainwright, 2020; Heard, 2021). Controls in prisons remained in place longer than in the general community given the high risk posed by the prison environment. Although at the time of writing (September 2023) many countries have eased restrictions within prisons and have entered a recovery phase, prisons in some countries have continued to implement strict restrictive measures (Sander & Jofré, 2023). Even in countries where restrictions have been lifted, it is possible that they may need to be reimposed in the future if Covid-19 outbreaks escalate or the context changes, for example the emergence of a variant of concern.

Despite an emerging knowledge base about the impact of the pandemic on the physical and mental health of people in prison, there is surprisingly little coherent primary research about the impact of the pandemic on the organisation and delivery of prison healthcare. Several authors have included only brief commentary on the delivery and receipt of prison healthcare, albeit situated in more expansive reports regarding the impact of Covid-19 on prisons and people in prison more generally (Edge et al., 2021; Gipson & Wainwright, 2020; Heard, 2021; Wainwright & Gipson, 2020). Recent evidence sourced from grey literature in England demonstrated that Covid-19 has led to a significant shift in the way that prison healthcare was delivered and received. Canvin and Sheard (2021) found that healthcare activity in the prison estate was disparate and depended upon local decision making. Healthcare services ceased completely at some prison sites but continued in varying degrees in others. Access to healthcare was often restricted to only urgent care or when there was a significant risk to a person’s life or long-term health. Mode of delivery was rapidly reconfigured with a move away from face-to-face appointments and towards telephone appointments or care through the cell door. Canvin and Sheard (2021) conclude that the risk of harm to people in prison was increased due to a reduction in the availability of healthcare and, in some instances, the needs of individuals were not met which resulted in direct harm.

We undertook a scoping review of the peer-reviewed academic literature regarding the impact of Covid-19 on the delivery and receipt of prison healthcare. We were cognisant of the potential lack of peer-reviewed literature if limiting our focus to just English prisons, and therefore broadened our search to be international. This scoping review is part of a wider study funded by the Economic and Social Research Council as part of the UK Research and Innovation’s rapid response to Covid-19, which explores the impact of the pandemic upon prison healthcare. The project team have a) conducted a qualitative study with 45 people from the following groups: those who have been in prison, prison healthcare staff and prison decision makers, b) performed a statistical analysis on over 25,000 anonymised prison healthcare records from 13 prisons in England, and c) undertaken an environmental scan of grey literature in England (Canvin & Sheard, 2021). The aim of this paper is to answer the following research question: What is known about the impact of Covid-19 upon the delivery and receipt of prison healthcare internationally? Our definition of ‘prisons’ includes prisons themselves, as well as jails and young offender institutions.

## Methods

We followed the first five stages of Arksey and O’Malley’s (2005) framework for conducting a scoping review: identifying the research question, literature searching, selection of studies, charting the data, and collating and reporting the results.

### Eligibility criteria

#### Inclusion criteria

- Prisons, young offender institutions and jails in any country, all ages, male and female (from here on in collectively referred to as ‘prisons’)
- Any type of peer-reviewed literature, except literature reviews, whose primary focus was on the impact of Covid-19 on prison healthcare
- For empirical literature, the deployment of any type of research method (quantitative or qualitative)
- Published between December 2019 and January 2022
- English language
- International literature

#### Exclusion criteria

- Primary focus of the paper was not on the impact of Covid-19 on prison healthcare specifically (for example, papers about the impact of Covid-19 on prison regime or papers about people in prisons fears of catching Covid-19)
- Community criminal justice settings (i.e., probation hostels), forensic mental health settings, secure children’s homes, and immigration detention centres
- Papers from non-peer reviewed sources
- Papers pertaining to the impact of Covid-19 on non-healthcare service delivery in prisons (i.e., education, employment, religious worship)
- Papers published before December 2019 and after January 2022
- Non-English language

### Search terms

A search strategy containing specific search terms was developed by the review team in collaboration with an Information Specialist from the University of Leeds. The search terms that were used reflected two key concepts: Covid-19 and prisons. The search terms relating to the Covid-19 concept were informed by the UK Health and Security Agency *Finding the evidence: Coronavirus* document (UK Health & Security Agency, 2021). The search terms pertaining to the prison concept were adapted from previous literature reviews focusing on prison settings (Bagnall et al., 2015; Wright et al., 2011). The terms were searched as key words, topics, Medical Subject Headings (MeSH) and subject headings, and terms were truncated where possible. Boolean operators were used to combine synonyms in the concept groups with OR prior to combing the two groups of search results with AND (see Additional File 1 for example of search terms used).

### Electronic databases searched

The search strategy was executed in the following databases: MEDLINE, CINAHL, Web of Science, PsycINFO, Embase and Criminal Justice Abstracts. These databases were selected on account of being highly relevant to the topics under investigation and, therefore, likely to retrieve literature meeting the inclusion criteria. The initial searches were executed on 24 June 2021. The searches were run again on 17 January 2022 to capture any new papers pertinent to the review question that had been published between execution of the initial searches and prior to submission of this scoping review for peer-review.

### Hand searches

To complement the electronic database search, a hand search of the following key journals was undertaken by PH: *International Journal of Prisoner Health, Journal of Correctional Health Care, Prison Service Journal, The Prison Journal*, *British Journal of General Practice* and a special edition of *Victims and Offenders* focusing on the global response of Covid-19 outbreaks in criminal justice settings. The reference lists of the 12 papers included in the scoping review were also searched, as were the reference lists of literature reviews that were excluded.

### Study selection

Two researchers (SB and PH) undertook the initial screening of the titles and corresponding abstracts retrieved from the electronic database searches and the hand searches, assessing these against the eligibility criteria to determine whether the articles should be retrieved for full-text review. For those articles identified for full-text review, the full-texts were retrieved and then reviewed by two researchers (SH and PH) to determine if the article was to be included in the final review; this was again assessed using the inclusion/exclusion criteria. Any discrepancies or ambiguities at both stages were resolved through discussion with a third reviewer (KC).

### Data extraction and analysis

Data from articles included in the review were extracted into a data charting table developed by three researchers on the review team (KC, SH and PH). The table was developed to enable direct comparison of included papers. Data fields included in the extraction table comprised the following: authors, year of publication, article title, source, country, study design/publication type, population, healthcare services affected, and key findings/recommendations. Two researchers (PH and SH) undertook the data extraction, whilst one researcher (PH) utilised thematic analysis to identify common themes across the included articles.

## Findings

**Fig. 1:**
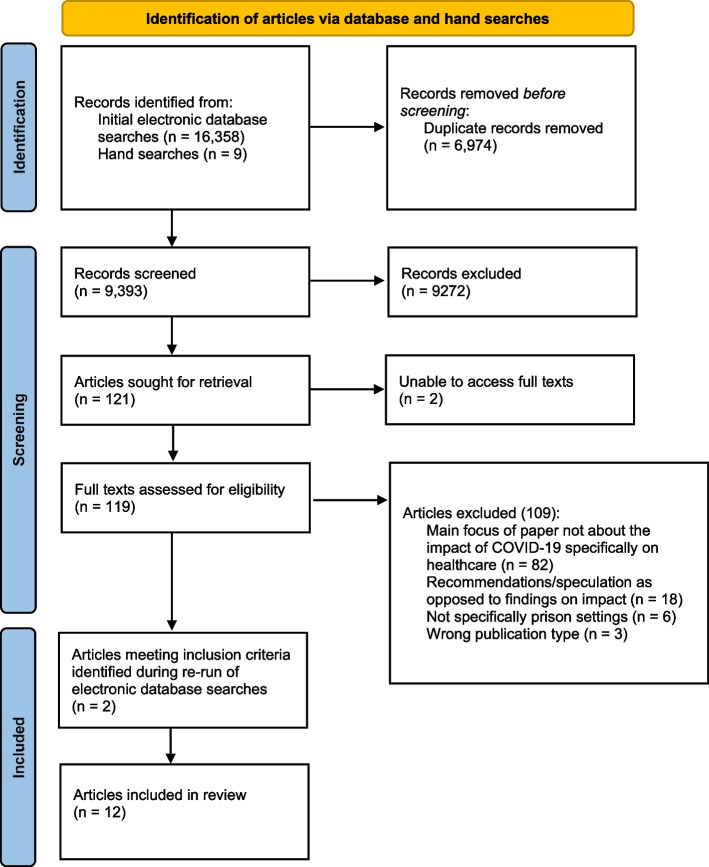
PRISMA flowchart documenting selection of evidence for scoping review

The initial searches of the electronic databases on 24 June 2021 returned 16,358 records, whilst the hand search returned a further nine. De-duplication was undertaken, removing 6,974 duplicate records. Initial screening of the abstracts and corresponding titles against the eligibility criteria of the remaining 9,393 records was undertaken, resulting in 121 records being identified for in-depth full-text review, two of which could not be obtained by the research team due to lack of access. Following the in-depth review of the 119 full-texts, 109 articles were subsequently excluded, leaving 10 which were included in this review. Following the re-run of the electronic database searches on 17 January 2022, a further two papers meeting the inclusion criteria were identified and were incorporated into the review. The PRISMA diagram below summarises the above process (Figure 1).

The characteristics of the included papers and their main findings are summarised in Table 1. Most of the articles focused on prisons in the USA (4), with the remainder exploring the impact of Covid-19 on prison healthcare services in England (2), Australia (2) Canada (1), France (1) and Italy (1), and one paper focused on 15 prisons across multiple countries in Europe (14 high income countries, and one upper middle-income country). In terms of the prison healthcare services affected by Covid-19, the majority of papers explored the impact on drug treatment services (5), followed by mental health (2), prison health screening services (2) and telemedicine (1); one paper discussed the impact on multiple health services, whilst another focused on the impact on a democratic therapeutic community prison. The majority of the articles were case studies (6). The remainder comprised two secondary data analyses, one cross-sectional study, one mixed-methods study, one commentary and one letter to the editor. We noticed that the findings of the papers fell into two distinct categories: healthcare disruption and healthcare innovation/adaption due to Covid-19. Therefore, we discuss the findings in relation to the following overarching questions: 1) how, and in what ways, was prison healthcare disrupted by Covid-19? and 2) how did prison healthcare innovate or adapt to respond to the constraints brought about by the pandemic?

**Table 1:** Articles included in the review

Author(s)/Year of publication	Title/Journal	Country/International	Study design/Publication type	Population described or studied	Service(s) affected	Key findings and recommendations
Akerman et al. (2020)	Social distancing in a social therapy environment – Therapeutic Communities: The International Journal of Therapeutic Communities	England	Case study	Male prisoners residing in HMP Grendon	Whole prison therapeutic community	Whole community meetings ceased; small group meetings and one-to-one meetings continued but in line with social distancing guidelines. The clinical focus of meetings changed, focusing on the here and now rather than the more diverse topics usually discussed. Distraction packs and daily bulletins distributed to residents. Impact likened to being in a mainstream prison as opposed to a therapeutic community environment, with a loss of sense of community reported. Non-operational staff utilised on the wings to support residents, with some wings introducing daily check-ins. Authors acknowledge that the return to ‘normality’ may be more difficult for therapeutic community prisons than mainstream prisons
Bandara et al. (2020)	Early effects of COVID-19 on programs providing medications for opioid use disorder in jails and prisons – Journal of Addiction Medicine	USA	Cross-sectional study	16 prisons/jails in the USA that were providing methadone/and or buprenorphine to inmates prior to the COVID-19 pandemic	Opioid substitution services	Survey responses demonstrated that 10/16 institutions reported to having downsized their opioid substitution programmes in response to COVID-19. 7/16 institutions reported that alterations to medication dispensing processes were made. 13/16 institutions reported using the same processes to make community follow-up appointments; 2 changed their processes and 1 discontinued scheduling follow-ups. 13/16 institutions reported that some programme participants were released earlier than scheduled through early release schemes. The authors propose that the use of telehealth may facilitate provision of opioid treatment services provided by community providers in jails/prisons through removing the need for face-to-face contact, and also that telehealth may increase access to community services for prisoners on release
Bartlett et al. (2021)	Hepatitis C screening and diagnosis in a Canadian provincial correctional system during the COVID-19 pandemic – Hepatology	Canada	Secondary data analysis	10 British Columbia Provincial Correctional Centres	Blood-borne virus screening – Hepatitis C Virus (HCV) screening/diagnosis	Compared to the first quarter of 2020 (i.e., pre-pandemic), there was a decrease in the number of HCV antibody (66%), RNA (67%) and genotype (68%) tests ordered by British Columbia Correctional Centres in the 2^nd^ quarter of 2020 (i.e., following declaration of the Covid-19 pandemic). However, the total number of HCV tests ordered as a proportion of new receptions into the facilities increased from 17% in 2019 to 23% in 2020
Blogg et al. (2021)	Lessons learned from keeping NSW’s prisons COVID-free – International Journal of Prisoner Health	Australia	Case study	Prisons	Various healthcare services	Youth Justice facilities provided ‘mental health’ (distraction) packs to prisoners. Due to the reduced illicit supply of drugs into prisons, there was an increased demand for opioid substitution services – in response, the depot buprenorphine programme was quickly scaled up. Mental health services continued to be provided – ‘at risk’ prisoners had regular mental health reviews via telemedicine, and, for all other prisoners, mental health services continued to be available, facilitated again by telemedicine. Throughout the course of the pandemic, telemedicine access increased by over 30%. Whilst movements of prisoners between centres was generally curtailed, movements relating to health needs continued
Burton et al. (2021)	Mental health services in a U.S. prison during the COVID-19 pandemic – Psychiatric Services	USA	Case study	1 US prison	Mental health services	Temporary mental health units were set up and there was a reassignment of mental health staff roles to deal with the increased demand for mental health services following the curtailment of prison transfers. Almost all mental health services provided in the prison implemented physical distancing measures. Group therapy sessions were impacted through a reduction in group sizes. Increased use of videoconferencing for psychiatric legal hearings and telepsychiatry became a key feature of care – by the end of March 2020, approximately 2/3 of all psychiatric consultations utilised telehealth. Face-to-face consultations still took place where indicated (i.e., where clinically indicated by a clinician). Both staff and patients seem satisfied with telepsychiatry services, and the authors acknowledge the benefits in terms of its efficiency
di Giacomo et al. (2020)	Italian prisons during the COVID-19 outbreak – American Journal of Public Health	Italy	Case study	1 Italian prison	Mental health services	Increased mental health service provision was provided, however weekly therapeutic groups were suspended. Authors report a similar level of mental health consultations in the first quarter of 2020 to the first quarter of 2019
Donelan et al. (2021)	COVID-19 and treating incarcerated populations for opioid use disorder – Journal of Substance Abuse Treatment	USA	Case study	1 US jail	Opioid substitution services	Opioid substitution services were adapted rapidly in response to the COVID-19 pandemic; provision of services moved away from groups to housing units, behavioural groups were cancelled, and medications were dispensed at cell-doors for those medically quarantining. Telehealth has also become increasingly used; to provide psychosocial support to distressed individuals, to provide in-reach services by community providers and for post-release re-entry programming. Early release schemes posed problems for the programme, as many programme participants were being released at short notice, creating difficulties with community care continuation planning. In response to this, a bespoke model was implemented, which relied heavily upon telehealth. The jail also collaborated with other agencies to facilitate take home doses for those being released from custody
Duncan et al. (2021)	Adaptations to jail-based buprenorphine treatment during the COVID-19 pandemic – Journal of Substance Abuse Treatment	USA	Case study	1 US jail	Opioid substitution services	Delivery of the existing opioid substitution programme continued during COVID-19. The relaxation of federal regulations meant that telemedicine could be implemented, leading to adaptations to the existing programme; buprenorphine initiation consultations were conducted via telemedicine as opposed to face-to-face and patients were offered buprenorphine taper on admission. Telemedicine was also used in other health areas (i.e., mental health and general health services). Adaptations to the nurse intake procedure were also made. The authors acknowledge that the reduced jail population in response to COVID-19 has aided access for patients to opioid substitution services. The authors suggest that telemedicine buprenorphine initiation should continue post-pandemic
Edge et al. (2020)	COVID-19: digital equivalence of health care in English prisons – The Lancet Digital Health	England	Commentary	Prisons	Prison telemedicine	HMPPS supported legislation changes enabling use of 4G-enabled tablets within prisons to combat connectivity issues previously experienced; proposed that all prisons within England will have 4G-enabled tablets, access to telemedicine and mobile access to patients’ electronic health records as a result of the pandemic. Due to implementation barriers, tablets were not implemented before the peak of the first wave of the pandemic ended. The authors acknowledge the many benefits of telemedicine for prisons (i.e., access to specialist services, reduced referral waiting times etc.) and highlight the need for HMPPS to keep up to date with new technological advances to ensure digital inequalities are not further widened
Montanari et al. (2021)	The impact of the COVID-19 lockdown on drug service provision in European prisons – International Journal of Prisoner Health	Europe	Mixed-methods study	Prisons	Drug treatment services	Most of the 15 European countries reported decreases in drug treatment/harm reduction service provision at the beginning of the pandemic. Services most affected by discontinuation or reduction were; interventions involving groups, interventions delivered by external providers, one-to-one psychosocial counselling, links to care in the community, preparations for release and therapeutic communities. Most countries reported no changes in service provision for; drug assessment at reception/intake, detoxification, opioid substitution initiation/maintenance, testing/treatment for blood-borne viruses, condom distribution, needle exchange programmes, overdose prevention advice and drug testing. Many countries reported innovative adaptations to services, such as consultations via telemedicine, auto-renewal of opioid substitution prescriptions, alteration to medication dispensing, and sharing of knowledge between countries. Some countries specifically acknowledged the importance of collaboration/communication between different agencies involved (i.e., prison service, health service, non-governmental/voluntary organisations)
Remy et al. (2021)	Lack of COVID-19 impact on managing Hepatitis C in prison like the general population – Hepatology	France	Secondary data analysis	Prisons	Blood-borne virus screening – HCV screening/diagnosis	After the introduction of the first French lockdown in 2020, between June and December 2020, HCV screening and treatment rates increased. There was no decrease in HCV-RNA tests and HCV screening in 2020 compared to 2019. All prisoners found to have detectable HCV RNA in 2020 were treated
Roberts et al. (2021)	Rapid upscale of depot buprenorphine (CAM2038) in custodial settings during the early COVID-19 pandemic in New South Wales, Australia – Addiction	Australia	Letter to Editor	Prisons	Opioid substitution services	To reduce risk of COVID-19 infection and reduce demand on resources, patients on sublingual buprenorphine-naloxone were switched to monthly buprenorphine depot. Authors acknowledge that collaboration was key to success of scaling up depot buprenorphine provision

### How did Covid-19 lead to a disruption of prison healthcare and which services were impacted?

#### Drug treatment services

Most of the papers comprising this scoping review focused on the impact that Covid-19 had on drug treatment services within prison settings, with the literature reporting an impact on most of these services in some way. There were reports of increased demand for prison drug treatment services in prisons in Europe and Australia, particularly for opioid substitution therapy (Blogg et al., 2021; Montanari et al., 2021; Roberts et al., 2021). There were reports of reductions or downsizing of drug treatment/harm reduction services in some US and European institutions (Bandara et al., 2020; Montanari et al., 2021), whilst in contrast, there were rapid upscales of programmes in New South Wales, Australia, specifically in the form of increased buprenorphine depot provision (i.e., an injection formulation of buprenorphine which releases slowly over time) (Blogg et al., 2021; Roberts et al., 2021). The rationale behind the drive regarding buprenorphine depot provision was that monthly dosing of such medication, as opposed to daily dosing of sublingual buprenorphine preparations, would significantly reduce infection risk and also reduce the burden on limited staff resources (Roberts et al., 2021). Indeed, in the study by Bandara et al. (2020) that reported downsizing of opioid substitution programmes, one of the main challenges reported by institutions was having adequate staff available to maintain delivery of the treatment.

It was evident that some aspects of drug treatment provision appeared to be more severely affected than others. For instance, Montanari et al. (2021), in their analysis of drug treatment services in prisons across 15 European countries, noted that most countries reported no changes with regards to provision of the following: drug assessment at reception/intake, detoxification, opioid substitution initiation/maintenance, testing/treatment for blood-borne viruses, condom distribution, needle exchange programmes, overdose prevention advice, and drug testing. In contrast, behavioural group therapies, which by their nature bring individuals together, were discontinued altogether or reduced in scale (Donelan et al., 2021; Montanari et al., 2021). Other drug treatment services consistently reported to have been impacted, either by discontinuation or reduction, were interventions delivered by external providers, one-to-one psychosocial counselling, therapeutic communities, links to care in the community, and preparations for release (Bandara et al., 2020; Donelan et al., 2021; Montanari et al., 2021). The latter two “through the gate services” were significantly disrupted by Covid-19 and this appeared to be two-fold. Firstly, some institutions released those receiving drug treatment early through early/rapid release schemes implemented to try and reduce the population being held in custody, and thus limit the spread of the virus (Bandara et al., 2020; Donelan et al., 2021). However, this often led to people in prison being released on short notice, which created difficulties with regards to prison healthcare workers arranging community care drug treatment follow-up (Donelan et al., 2021). Indeed, some institutions reported to adapting processes for linking people with community care providers (Bandara et al., 2020; Donelan et al., 2021), whilst others discontinued such linkage altogether (Bandara et al., 2020; Montanari et al., 2021). Secondly, Montanari et al. (2021) found that the withdrawal/reduction of external care providers, which many countries reported, also had a detrimental impact upon linkage to community care and release planning.

#### Mental health services

In general, the papers in this review reported that access to mental health services have been available to people in prison throughout the pandemic. However, there has been a shift in how such services are being delivered, with many consultations now taking place via telemedicine or telephone, as opposed to in-person (Blogg et al., 2021; Burton et al., 2021). Where the Covid-19 pandemic does appear to have had a particularly detrimental impact on prison mental health services is with regards to group therapies, with reports of therapeutic groups/community meetings either being significantly reduced or ceased altogether (Akerman et al., 2020; Burton et al., 2021; di Giacomo et al., 2020). This appeared to be especially problematic for the therapeutic community prison, where the cessation of behavioural group work and community meetings were suggested to have affected residents’ therapeutic work, and made the prison feel more ‘mainstream’, as opposed to a therapeutic community environment (Akerman et al., 2020).

#### Screening services

Two studies explored the impact of Covid-19 on prison healthcare screening services, with both focusing specifically on the impact on Hepatitis C (HCV) screening. Whilst Remy et al. (2021) found that HCV screening rates in France did not decrease in 2020 from 2019, Bartlett et al. (2021) found conflicting results in Canada, where the number of HCV antibody, RNA and genotype tests ordered decreased from the first quarter of 2020 (pre-pandemic) to the second quarter of 2020 (during the pandemic), suggesting Covid-19 had a detrimental effect on such an important screening service. However, when examining the total number of HCV screening tests ordered as a proportion of new receptions into the 10 provincial prisons in Canada, the authors found that the number of tests ordered actually increased from 17% in 2019 to 23% in 2020 (Bartlett et al., 2021). Thus, the decrease in screening tests ordered in the second quarter of 2020 may be explained by a reduced number of individuals entering prisons as opposed to a detrimental impact on the quality of screening services resulting from the Covid-19 pandemic.

### How did Covid-19 lead to innovations or other positive adaptions to prison healthcare?

#### Digital solutions

One of the main innovations in prison healthcare practice was the increased use of telehealth (i.e., the use of digital platforms, such as telephone and video-calls, by a clinician to diagnose and treat patients) to ensure continued service delivery during the Covid-19 pandemic. Such digital platforms were utilised to support continued provision of mental health services (Blogg et al., 2021; Burton et al., 2021), drug treatment services (Donelan et al., 2021; Duncan et al., 2021; Montanari et al., 2021), and general healthcare provision (Duncan et al., 2021). For instance, Burton et al. (2021) reported that 60% of all psychiatric encounters conducted by the end of March 2020 in a prison in the USA were done so using telepsychiatry technology. In the case of drug treatment services, some institutions reported using telehealth specifically to ensure continuity of re-entry services and external service provider provision (Donelan et al., 2021), which as noted above were aspects of drug treatment services reported to be significantly hindered by the Covid-19 pandemic. For example, in one jail in the USA, re-entry services switched to using telehealth platforms to enable people being released from custody to be linked with drug treatment providers in the community (Donelan et al., 2021). Additionally, the jail utilised telehealth to provide in-reach services by external providers who were unable to physically access the jail on account of restricted access in response to the pandemic (Donelan et al., 2021).

In the USA and England, relaxation of federal regulations (Bandara et al., 2020; Duncan et al., 2021) and legislation changes (Edge et al., 2020) respectively were reported to have been introduced to support digital innovations in healthcare provision in response to Covid-19. For instance, in the USA, prior to the pandemic, initial buprenorphine prescriptions were only permitted following a face-to-face consultation with a licenced prescriber. However, in response to the Covid-19 pandemic, relaxations to such practices were implemented, allowing prescribers to initiate buprenorphine prescriptions following telehealth appointments. These amendments to regulations have enabled prison settings to adapt buprenorphine initiation prescribing practices so that these are now able to take place remotely as opposed to in-person (Bandara et al., 2020; Duncan et al., 2021). This allows prescribers to consult patients in a timely manner whilst also minimising risk of infection due to physical distancing protocols being in place (Duncan et al., 2021). Other digital solutions utilised in some prisons in Europe throughout the pandemic have been auto-renewals of opioid substitute medications (France) and the undertaking of training activities (Italy) (Montanari et al., 2021). Whilst digital solutions have been beneficial in supporting the continued provision of healthcare services in prison settings, some authors have acknowledged that such increases in telehealth capacity have only been enabled through increased financial support (Blogg et al., 2021; Donelan et al., 2021; Edge et al., 2020), for example through emergency/grant funding or re-direction of existing funds not being utilised due to the restrictions put in place at establishments.

#### Non-digital adaptations

As well as digital innovations, articles reported that prison settings rapidly implemented other adaptations to healthcare services in response to the pandemic, one of which was changes to medication prescribing and dispensing. In New South Wales, Australia, people in prison being prescribed buprenorphine-naloxone in sublingual form were switched to buprenorphine depot due to its advantages compared with other forms of opioid substitution medications discussed earlier (Roberts et al., 2021). Other medication prescribing adaptations reported in some US institutes included provision of take-home opioid substitute medication doses for people being released from jail (Donelan et al., 2021) and options to undergo a buprenorphine taper on admission to jail (Duncan et al., 2021). Some articles from both the USA and Europe reported of alterations to how medications were being dispensed to people in prison due to the pandemic (Bandara et al., 2020; Montanari et al., 2021). This primarily took the form of a change in the physical location that medications were being given to people in prison, for instance within cells as opposed to the usual location of the prison healthcare centre.

Articles reported that healthcare services had adapted to accommodate physical distancing measures, both patient-to-patient interactions, and also those between patients and staff. For instance, where in-person, one-to-one consultations between clinicians and people in prison took place, and in the very few instances where small mental health groups continued, these were reported to be conducted in line with the physical distancing guidelines in place at the establishment (i.e., those involved in the consultations/group being at least two metres apart from one another) (Akerman et al., 2020; Burton et al., 2021). Additionally, there were reports in Luxembourg of dividing glass barriers being installed to keep drug treatment clinicians and people in prison physically distanced during consultations (Montanari et al., 2021).

Due to the cancellation of therapeutic work and because of the increased likelihood of feelings of isolation due to lockdown measures, there were reports of in-cell therapeutic and diversionary materials being provided to people in prison. For instance, Donelan et al. (2021) reported people receiving drug treatment therapy being encouraged to work independently on recovery journals in the US, whilst in Australia and England, distraction packs covering aspects such as coping strategies and mindfulness techniques were distributed to people across the prison to support mental health (Akerman et al., 2020; Blogg et al., 2021).

One final adaptation reported was the reassignment of staff roles for existing members of staff working within custodial settings, particularly in the area of mental health. One prison in the USA reported to the reassignment of staff roles for clinicians already delivering mental health services to deal with the increased demand for inpatient psychiatric care resulting from the curtailment of transfers of people in prison between establishments because of Covid-19 (Burton et al., 2021). Another prison in England made the decision to utilise non-custodial staff on the prison wings to provide emotional support to the therapeutic community residents residing at the prison (Akerman et al., 2020).

### Collaboration

Some of the articles specifically discussed the importance of collaboration between stakeholders, including prison services, prison healthcare services, external providers etc., in ensuring ongoing healthcare delivery in prison settings during the pandemic (Blogg et al., 2021; Burton et al., 2021; Donelan et al., 2021; Montanari et al., 2021; Roberts et al., 2021). The setting up of telepsychiatry services in the USA (Burton et al., 2021), scale-up of buprenorphine depot in Australia (Roberts et al., 2021), and provision of opioid substitute medication for those being released from jail in the USA (Donelan et al., 2021) all acknowledged how partnership working had been key to delivering these vital services. There were also reports of collaborative working between countries, with Norway and the Czech Republic sharing drug treatment strategy responses to the unfolding Covid-19 situation (Montanari et al., 2021).

## Discussion

Our scoping review found that the Covid-19 pandemic disrupted some elements of prison healthcare in a demonstrable manner, and the peer-reviewed literature focused mainly on drug treatment services, with an additional but lesser focus on mental health provision and blood-borne virus screening. Conversely, we found that Covid-19 led to innovations or positive adaptations in the delivery of prison healthcare, with the main element of this being an increased use of telehealth, sometimes to maintain service provision. There were various non-digital innovations, which differed between countries.

We scoped the literature internationally and found that the prison-community interface and healthcare relationship between these two settings was disrupted in many high-income contexts across the world. Early release schemes were introduced in several countries to proactively save lives, particularly at the start of the pandemic. England lagged behind many other countries and by July 2020 had only conducted the early release of 80 people despite hopes of almost 15,000 people near the end of their sentence being released early (Edge et al., 2020). Donelan et al. (2021) found that early release schemes in the USA introduced problems as many people were released at short notice which created issues with care planning for their healthcare in the community, particularly in relation to onward opioid substitution prescribing. Healthcare planning prior to people being released from prison was further compounded by either the reduction or total withdrawal of external care providers entering the prisons due to the pandemic, as found by Montanari et al. (2021) in their reportage based on 15 European countries. This was highly disruptive to the prison-community relationship as external healthcare providers are primarily the providers that aid people in prison for release and link people to treatment in the community. The fact that “through the gate” services have been so affected by Covid-19 is of concern given that the first couple of weeks following release from custody is a particularly vulnerable time for individuals in terms of drugs overdose risk for those with a history of drug use (Merrall et al., 2010). A recent ‘Report to Prevent Future Deaths’ notice in the UK found that a break in continuity of opioid substitution treatment led to the death of a 41-year-old woman within a week of release from prison, after a prescription error slipped between the responsibility of a “through the gate” provider and a community pharmacy (Chipperfield, 2022). This emphasises the importance of these external services and how the impact of Covid-19 on imprisoned people has reverberated further than the prison gates.

We identified digital health, and more precisely telehealth solutions, as a major factor in ensuring the continuation of some prison healthcare services, such as mental health (Blogg et al., 2021; Burton et al., 2021) and drug treatment (Donelan et al., 2021; Duncan et al., 2021; Montanari et al., 2021). This was seen across Europe, Australia, and the USA. Edge et al. (2020) has discussed the digital inequality between prison healthcare and community healthcare in England and notes that prisons have a tradition of poor adoption of digital technologies. At the start of the pandemic, 50 out of 117 prison sites had a connectivity that was too poor for videoconferencing. Rapid legislation changes meant that 4G-enabled tablet computers could be used to provide telehealthcare, but implementation barriers meant that widespread use of tablet computers was not enacted until after the first wave of Covid-19 infections had peaked (Edge et al., 2020). It could be that outside of England, other countries have a starting point of use of digital technology in prisons that is more mature and further developed, hence entire healthcare programmes being able to switch to telehealth methods of delivery. Edge et al. (2022) have noted that the USA in particular have had historical success in implementing telehealth in prisons, suggesting that this may be because the US correctional system has overall responsibility for health budgets and is directly responsible for commissioning prison healthcare services. Overall, the published papers included in our review regarding telehealth and Covid-19 demonstrated that telehealth allowed a continued delivery of key services which may have been discontinued completely or scaled back in size without the use of such a digital solution. Coming full circle, we found that in a Massachusetts jail, telehealth solutions were used to ensure external provider provision for drug treatment services when these parties were not physically able to enter establishments because of the pandemic (Donelan et al., 2021). This seemed to be an outlier with the other published literature pointing to external provider services being largely stripped back, as discussed above.

At the time of writing, we did not find any peer-reviewed literature which looked at one specific aspect of healthcare across the whole of the prison system in one country or looked at one aspect of prison healthcare internationally. A study in Australia considered the impact of Covid-19 on various aspects of healthcare but this was in one region (New South Wales) (Blogg et al., 2021). Another study looked across 15 European countries but concentrated on drug treatment services (Montanari et al., 2021). We were only able to include two papers from the UK and both of these were focused on England specifically, with one being a case study about the therapeutic environment of a male prison (Akerman et al., 2020) and the other being a national level commentary about the state of play of prison telehealth as a consequence of the pandemic (Edge et al., 2020). As previously stated, most studies tended to focus on the changes to drug treatment services or mental health services. There was a distinct lack of peer-reviewed evidence surrounding routine aspects of physical healthcare, such as long-term conditions management, dentistry, podiatry, and screening programmes, apart from blood-borne viruses. An extensive amount of information about how Covid-19 impacted on prison healthcare is confined to the grey literature, such as prison inspection reports and briefings by charities/third sector organisations (Canvin & Sheard, 2021). Canvin and Sheard (2021) in their environmental scan of grey literature pertaining to English prisons found that dentistry, podiatry, physiotherapy, and non-urgent GP appointments were all affected by the pandemic, as access was reduced (dentistry reduced access was said to be “excessive”, and such a finding reflected community reports where dental services were suspended on account of the use of aerosol generating procedures in a setting of close proximity between dental clinicians and their patients (Trivedy et al., 2020)). It could be that the extensive lifecycle of the peer-review process has been an inhibiting factor in peer-reviewed evidence not yet coming to light about these areas of healthcare in contrast to the grey literature, whose authors are not inhibited from publishing their findings in a timely manner. Additionally, some countries placed restrictions on research studies taking place within prison establishments to minimise the risk of Covid-19 transmission, and this again may explain the lack of published peer-reviewed literature in this area. When undertaking our review, we found a large volume of peer-reviewed papers which considered the impact of Covid-19 on people in prison, often with a focus on their mental or physical health. Tangential reference (at the level of a few sentences) was sometimes made to prison healthcare delivery, but we only found 12 peer-reviewed papers internationally to date which had the sole focus of their study as the impact of Covid-19 on prison healthcare itself.

Whilst our scoping review was internationally focused, only papers from Europe, North America, and Australia met the inclusion criteria and were thus included in the review. Given that no papers exploring prisons in Africa, South America, and Asia were included, we are unable to draw conclusions about what impact Covid-19 had on prison healthcare delivery in these regions. This is a particular important point to note given the well documented fragility of prison healthcare systems in some low-middle income countries within these geographic regions, even prior to the pandemic (Arambulo et al., 2021; de Oliveira Andrade, 2020; Van Hout, 2020). For instance, in countries such as South Africa, Brazil and India, many prisoners are reliant on visiting relatives for the provision of medication and hygiene products (Heard, 2021).

### Strengths and limitations

A major strength of our scoping review is that it looked internationally to include peer-reviewed literature from across the globe. Furthermore, our inclusion criteria were expansive, and we included papers which focused upon any type of prison or jail, serving any gender or age. We also used a comprehensive search strategy involving numerous electronic databases which was complemented by a hand-search of key journals. The review had several limitations. Firstly, we did not appraise the quality of the papers we included as this was a scoping review rather than a systematic review. Second, we were unable to access some of the full texts of the articles identified through the electronic database search despite requests through multiple University libraries. Finally, articles not published in English were excluded due to time and financial constraints.

## Conclusion

Our scoping review highlighted that the literature pertaining to the impact of the Covid-19 pandemic upon high-income country prison healthcare delivery was largely confined to exploring either drug treatment or mental health service provision. There was a lack of published evidence pertaining to primary care provision and other routine services such as dentistry, physiotherapy, and podiatry, and thus these could be the focus of future research. Overall, the prison healthcare services reported on were disrupted by the pandemic but to differing extents. For instance, whilst behavioural group therapies and services provided by external organisations were scaled back significantly or ceased altogether, other services such as opiate maintenance therapy and screening for blood-borne viruses reported little or no changes. Prison healthcare adapted the way in which services were delivered to facilitate ongoing provision in a safe manner, for example through changes to the medication dispensing process, the introduction of physical distancing measures, and an increase in consultations taking place via telemedicine. Telemedicine, in particular, appears to have facilitated continuity of care which may have otherwise ceased, and thus its use in prisons, as we move forward out of the pandemic, should be considered.

### Supplementary Information

**Additional file 1.** Search used in the electronic databases

## Data Availability

Data sharing is not applicable to this article as no datasets were generated or analysed during the current study.
