# Supplementary material for: Understanding the impact of Covid-19 on the delivery and receipt of prison healthcare: an international scoping review
Source: Health Justice. 2023 Oct 17;11:42. doi: 10.1186/s40352-023-00242-9 (PMC10583455; doi:10.1186/s40352-023-00242-9)
Supplement: Supplementary file 1 — Additional file 1. Search used in the electronic databases [file 40352_2023_242_MOESM1_ESM.docx]

Additional File 1 – Search used in the electronic databases

| **Covid-19** | **Prisons** |
| --- | --- |
| coronavirus* | prison* |
| coronovirus* | imprison* |
| “Coronavirus" | secur* |
| "Coronavirus Infections" | incarcerat* |
| "Wuhan coronavirus" | Inmate* |
| Wuhan | offend* |
| "2019-nCoV" | Jail* |
| COVID-19 | detention |
| CORVID-19 | detain* |
| CONVID-19 | “secure estate” |
| WN-CoV | justice |
| HCoV-19 | confine* |
| "novel coronavirus" | penal |
| "new coronavirus" | penitentiary |
| CoV | “criminal justice” |
| 2019-novel | correction* |
| new coronavirus | guard |
| ncov | secure adj2 (unit or units or facility or institution* or facilities or centre* or center*) |
| SARS-CoV-2 | correctional adj2 (units or unit or facility or institution* or centre* or center* or system or facilities) |
| SARS-Cov-19 | “Ministry of Justice” |
| ncov*wuhan | Prisons/ |
| (outbreak* OR "respiratory illness" OR "respiratory disease" OR respiratory symptom* OR seafood market OR food market OR wildlife) and (Wuhan OR China OR Chinese) | Prisoners/ |
| “Hubei province virus” | Criminals/ |
| COVID-19/ |  |
| Coronavirus Infections/ |  |
| Coronavirus/ |  |
| betacoronavirus/ |  |
| SARS-CoV-2/ |  |
